# Holistic feedback approach with video and peer discussion under teacher supervision

**DOI:** 10.1186/s12909-017-1017-x

**Published:** 2017-09-29

**Authors:** Agra Dilshani Hunukumbure, Susan F Smith, Saroj Das

**Affiliations:** 1 Hillingdon Hospital, Pield Heath Road, Uxbridge, UB8 3NN UK; 2 Imperial College London, London, UK; 3 Medical Education Research Unit, Alexander Fleming Building, Imperial College London, South Kensington Campus, London, SW7 2AZ UK

**Keywords:** Feedback, Video, Self-reflection, Peer discussion, Teacher guidance, Holistic, Undergraduate

## Abstract

**Background:**

High quality feedback is vital to learning in medical education but many students and teachers have expressed dissatisfaction on current feedback practices. Lack of teachers’ insight into students’ feedback requirements may be a key, which might be addressed by giving control to the students with student led feedback practices. The conceptual framework was built on three dimensions of learning theory by Illeris and Vygotsky’s zone of proximal development and scaffolding. We introduced a feedback session with self-reflection and peer feedback in the form of open discussion on video-recorded student performances under teacher’s guidance. The aims of this qualitative study were to explore students’ perception on this holistic feedback approach and to investigate ways of maximising effective feedback and learning.

**Methods:**

Semi-structured interviews were used to gather data which were evaluated using a thematic analytical approach. The participants were third year medical students of Imperial College London on clinical placements at Hillingdon Hospital.

**Results:**

Video based self-reflection helped some students to identify mistakes in communication and technical skills of which they were unaware prior to the session. Those who were new to video feedback found their expected self-image different to that of their actual image on video, leading to some distress. However many also identified that mistakes were not unique to themselves through peer videos and learnt from both model performances and from each other’s mistakes. Balancing honest feedback with empathy was a challenge for many during peer discussion. The teacher played a vital role in making the session a success by providing guidance and a supportive environment.

**Conclusions:**

This study has demonstrated many potential benefits of this holistic feedback approach with video based self-reflection and peer discussion with students engaging at a deeper cognitive level than the standard descriptive feedback.

## Background

Feedback plays a crucial role in shaping a competent clinical practitioner [1]. In recent years many failures in providing effective feedback to medical students have been highlighted [2], with both students and teachers expressing dissatisfaction with the status quo. Delayed, overly brief and overly complex feedback are common student complaints [2, 3]. The teachers’ disappointment arises when students ignore feedback and repeat the uncorrected practice [2].

We believe teacher-dominated practice is one explanation for the ineffectiveness of feedback [4]. The expectations of students may differ to those of their teachers [5]. Feedback providers may have a substantial amount of knowledge and experience when compared with receivers [6], but teachers have limited insight into the receiver’s understanding, [5] which may result in feedback being beyond the receiver’s expertise to incorporate into their practice. Moving control of feedback from teacher to students may provide some answers.

**Fig. 1:**
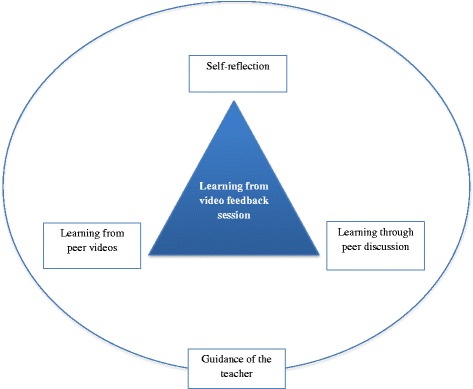
Feedback approach – Learning through self-reflection on videod performance, reflection on peer videos and peer discussion under the supervision of teacher

Self-reflection and peer feedback come from the students. Peers play a huge role in our lives and their contributions to the feedback process can be valuable [7]. We thought use of videos might facilitate self-reflection and peer feedback, whilst the presence of a teacher in these discussions might also be of help guiding the session, providing expert opinion and addressing any inaccuracy in students’ comments. We designed a feedback approach incorporating these concepts (Fig. 1), choosing three dimensions of learning theory by Illeris to form the conceptual framework for our approach. This theory describes a holistic approach encompassing all cognitive, psychological and social aspects of learning [8]. The video feedback session aligns well with these three perspectives facilitating a comprehensive feedback approach. Illeris’s theory does not elaborate on collaborative learning. We referred to Vygotsky’s concept of zone of proximal development and scaffolding to underpin the peer discussion [9]. A teacher or more able peer can help where an individual struggles. In the feedback session, almost all the students have achieved a certain level of competency, but each student may possess specific advanced knowledge, better understanding or awareness of resources, so the role of the more able peer can constantly shift between individuals. Therefore each person has some contribution, but as a group they can co-construct their understanding and have the potential to achieve a higher level than individually.

### Video and self-reflection

Video provides a window for students to self-reflect on their performances and creates an opportunity for evidence-based reflection on an authentic setting rather than from memory, which may not capture the true performance [10]. According to Creer and Miklich as cited by Dowrick [10] viewing one’s role play has a more powerful impact on subsequent improvement than the role play itself. A number of studies have demonstrated the improvement gained by students in terms of their practical and communications skills particularly non-verbal skills through the use of video feedback [11, 12]. The main drawbacks reported of videos were initial anxiety, public self-awareness and students being overly self-critical [13, 14].

### Peer videos and learning

In this study we considered peers as those who are in the same year group, have a similar exposure in clinical practice and have completed the same OSCE (Objective Structured Clinical Examination) circuit. Taking up an assessor’s role with the aim of giving feedback may promote the development of greater objectivity towards assessment criteria [6, 7]. As each student can approximate him/herself closely to others in the group, peer videos can promote self-reflection and metacognitive skills [15]. Peers’ comments and questioning can further enhance self-reflection and deepen understanding.

### Peer feedback and discussion

We adopted the definition for peer feedback as ‘a communication process through which learners enter into dialogues related to performance and standards’ [6]. The students are responsible for their contributions but through group discussion their understanding gains breadth as they encounter different explanations, opinions and techniques. It may also gain depth through dialogue connecting with understanding acquired in pre-clinical years. As they contest their views with peers they become confident about their knowledge and understanding [16, 17]. Thus the core of this feedback exercise is free and open contribution from each individual to generate a vibrant discussion. Discussing feedback in a group setting can be challenging compared with feedback being given individually [6, 16, 18] and requires the students to work collaboratively sharing their knowledge, skills and understanding.

### Teacher’s role

Peer discussion can create a window for the teacher to learn an individual’s thoughts from self-reflection whilst gaining insight into other group members’ understanding through their contributions to the discussion. The expert knowledge and these insights combined can guide the teacher to provide students with more personalised and meaningful feedback.

### Aims of the study

The aims of this study were to explore the practical applicability of this concept of holistic feedback approach and investigate the benefits and challenges through the perceptions of medical students and to look at ways of maximising effective feedback and learning.

## Methods

### Context

At Imperial College London, the third year of medical study is the first full clinical year and consists of three ten week placements. At the end of each placement, students have a formative five station OSCE. We have incorporated the feedback approach into this assessment. 34 students, in six groups of five or six, completed the OSCE. Each student within the group was videoed on a different station. 28 students attended the feedback session.

### Why a formative OSCE?

The OSCE is a practical examination, providing the opportunity to embed many practical skills, giving the opportunity to explore the applicability of the feedback session on different areas. Secondly the students will prepare and are likely to perform to the best of their ability in assessment settings, making the feedback more effective. Thirdly the main aim of formative assessment is feedback [19] and through a session like this, we can make the best use of this opportunity.

### Feedback session

All students receive training in giving and receiving constructive feedback in the early years of their programme, in communication skills and problem based learning. Because of this, we did not provide any additional instruction on the provision of feedback. The discussion started with an introduction by the teacher outlining the format of the session. After each video clip, the featured student was encouraged to self-reflect, with teacher prompting if necessary. Then peers were asked to contribute to the discussion sharing their experiences, any doubts/gaps in their knowledge or understanding and providing constructive feedback to their colleague. No scoring checklists were provided to encourage reflection and open spontaneous discussion within the group. The students were given the choice of withholding their videos from the group if they had performed badly in a station as judged by the teacher and one student of the 28 exercised this option and received private feedback from a tutor.

### Study design

A social constructionist view was adopted in designing the study. Semi-structured interviews were used to explore each student’s perspective individually [20]. We believed it would be easier for the students to be honest when they were interviewed individually especially on sensitive areas such as negative peer feedback.

In this study one of the authors held a dual position as the teacher as well as the researcher. Being a teacher in this context may be helpful in making meaning. From a social constructionist point of view, this helped to co-construct the knowledge with the participants. On the other hand it could bring bias to the study. However, Lichtman argues that: ‘Researchers should not strive to be objective and look for ways to reduce bias. Rather, they need to face head on the subjective nature of their role’ [21]. Therefore we believe that the context of the researcher in relation to the study is important and thus revealing it explicitly is essential. In this study, the researcher consciously adopted a neutral personal stance exploring the benefits and challenges in the feedback sessions.

### Participants

The students were recruited on a voluntary basis from those attached to Hillingdon Hospital in February 2014 who had participated in the formative assessment and feedback session. Seven students (four males and three females, aged 21–28 years) participated in the study. Participants came from a variety of backgrounds including graduate entrants and international students.

### Data analysis

The interviews were transcribed and the data analysed using a thematic analytical approach [20]. In the transcript, participants were anonymised by number and letter, M for males and F for females.

As described by Strauss and Corbin, open codes were generated and were grouped into broad themes [22]. Folders were created for each theme electronically in order to handle the vast amount of data generated through the interviews. Afterwards, in each folder, open codes were grouped together producing axial codes which consists of many categories and subcategories using highlight colours in Microsoft word and font colours. Similar colour codes were cut and pasted together in order to analyse further into similarities and differences of opinions. According to Strauss and Corbin: ‘open coding and the use it makes of questioning and constant comparison enable investigators to break through subjectivity and bias’ [22].

### Research ethics

Ethical approval was granted by Medical Education Ethic Committee of Imperial College (MEEC 1314–11). We also obtained permission from the research and development department of Hillingdon Hospital.

## Results

The core of this holistic feedback approach was learning through feedback in a group setting with teacher supervision. The results were analysed in this context, under these main themes (as illustrated in Fig. 1); exploring individual learning from video based self-reflection, mutual learning from peer videos, peer discussion and teacher’s contribution.

### Video and self-reflection

The videos provided an authentic picture of the students’ performances without relying on memory, facilitating self-reflection.

“I thought I really managed to put compassion across and then when I watched the video…sound really stony and that was quite weird for me”. (F2).

“When I was suturing, I knew to not touch the needle and make the proper sterile field but what I was actually doing, was completely different and when I watched the video, it made me realised that , that there was big disparity”. (F2).

Some students were unaware of their mistakes. This may explain why they disregard teachers’ feedback. Some were fixated on mistakes, denting their confidence.

“I had forgotten what part of the examination came after that part,… I thought, I had been thinking for ages about what I was going to do next and I was panicking,…it seemed like a really long time to me but when I watched it back it wasn't really even that noticeable”. (F7).

Another interesting finding was students’ appreciation of videos as a tool for reflection. M4 did not believe learning from videos.

“For me, I didn’t find it overly useful because I feel like, I remember actual, what I thought I did wrong in the session”. M4.

After teacher facilitated self-reflection:

“The problem with watching yourself is strange, I do weird things with my hands, I cannot talk without using my hands, probably be best to have my hands at my side and see if I can learn to talk without pointing my hands at peoples face and gesticulating strange movements”. M4.

His response reflected that he identified the learning need on his non-verbal communications skills, though he did not recognise this as learning.

**Fig. 2:**
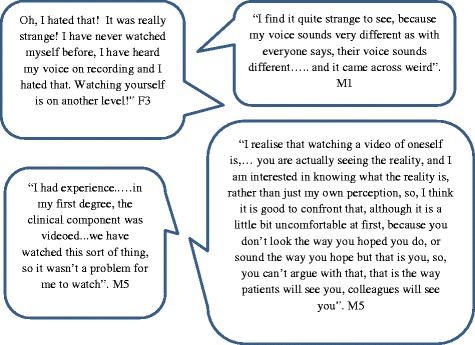
Students’ perceptions on challenges to video based self-reflection

The main challenge identified on videos was the anxiety of watching own self (Fig. 2) when their expected self-image was different to that of their actual image.

Many students overcame their initial anxiety to varying degrees during the feedback session and managed to look at their videos objectively. Those who had previous exposure to videos were less apprehensive such as M5, a graduate entry student who had video feedback regularly during his first degree.

### Mutual learning from peer videos

**Fig. 3:**
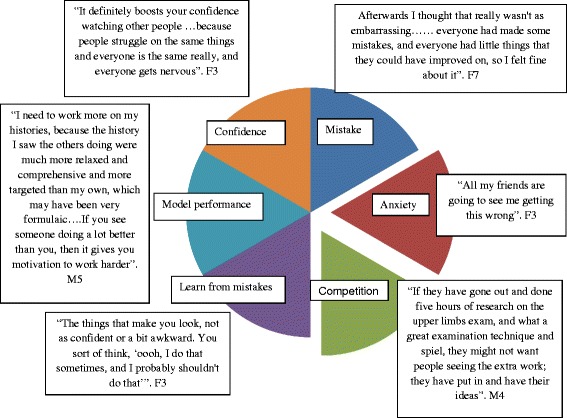
The students’ perceptions on peer videos, demonstrating advantages and challenges

Many students expressed initial apprehension about watching videos in a group, worrying about sharing their mistakes. The session helped them realise that every student had short-comings. This helped change their perception and improve their self-confidence (Fig. 3).

Our study showed that observing peer videos had a positive influence on students regardless of whether it was a better or worse performance than their own. When the performance was better it acted as a model demonstration and many students were motivated to work hard to achieve similar status. If it was lower, the students reflected on their own performances through other’s mistakes and made a point to avoid them in the future.

### Reactions to peer mistakes

Some students expressed empathy and understanding towards peers’ mistakes, which was an essential element for healthy peer discussion. They were forthcoming with their own experiences as well as useful practical tips and learning resources:

“I would be a bit sorry for them, and a bit embarrassed for them, that everyone had seen….. some silly mistake, they made. I don't think that it would make me think any less of them, or anything as we all make mistakes”. F7.

In contrast, M4 had different views:

“If it was someone I knew in the context who does really well, or normally breezes through OSCE stations, and if I see them make mistakes, it brings everyone down to a human level, and makes everyone aware of the fact that no one is perfect and we are still learning”.

“They should just be your friends... but there is an element, that you can't really help judging someone. I think it’s quite nasty and uncomfortable having to watch someone do something badly, and then feeling bad because…. you are automatically feeling a slight superiority, if you did better on that station. Especially if you are naturally competitive and competition is something that drives you”.

Both these quotes were made by M4 on performances with mistakes; interestingly his interpretation depended on the person rather than the performance, suggesting that some students have established attitudes about their colleagues well before these sessions. Although M4 admitted to these thoughts, his feedback in the class was supportive.

### Peer discussion

We identified from the literature [6, 18] that peer feedback and collaborative learning can be challenging. Thus our focus was to investigate what hinders open and honest discussion among peers.

### Lack of experience

Though students have had teaching on giving feedback during the first two years, they still find giving feedback difficult.

### ‘Feedback’

The word ‘feedback’ may have different meanings to different people. Many participants shared a common understanding which was a combination of positive comments enforcing correct behaviours and constructive criticism to correct mistakes. However some students had different views:

“If you tell someone to watch a video and give feedback, you are watching for the mistakes, you are watching for all the negative things, you are waiting for them to slip up”. M4.

### Honest feedback

**Fig. 4:**
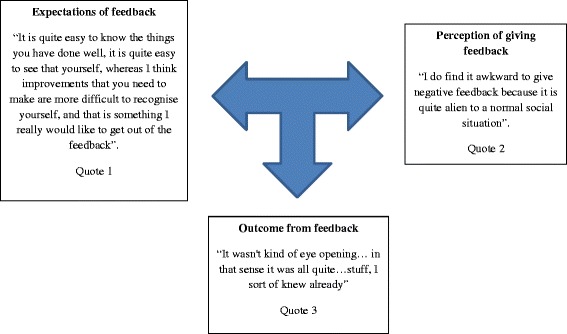
Feedback dilemma

Some participants found it hard to give honest feedback to their colleagues. We chose one participant, F7 as an example and compared her expectations of receiving honest feedback to that of her perception into giving feedback and end results, the outcome (Fig. 4).

All participants’ unanimous expectation of feedback session was to get constructive feedback and improve their performances. Nevertheless some contradicted own views when giving feedback to their peers which were biased towards positivity. Underpinning views included the fear of upsetting relationships among peers and the unwillingness to bring up mistakes in conversation. F7’s second quotation unravels powerfully established views in our society when commenting on another. The consequence was ineffective feedback, resulting in F7 expressing her frustration at failing to receive constructive feedback to improve her performance.

### What may help towards honest feedback?

Having identified that this is a challenge for many students, we looked at the factors that helped students to be honest with their feedback.

Some participants used each other’s response as a guide to determine own strategy. Both quotes from below demonstrate that the students have made an effort in giving honest feedback.

“You think someone has taken the time to come up with some improvements that you could make,……. when someone has obviously put thought into it, rather than saying ‘Ya that was good’, then it kind of makes you really watch what they are doing closely, and you can give really productive feedback”. F7.

“If someone was more forthcoming with their comments, then that would help me be more forthcoming when commenting on them. So I would think they are one of these people who are comfortable giving feedback, so I would assume they are comfortable to receive it as well”. F3.

The first quote indicated F7’s appreciation of constructive feedback from her peers. It also revealed that she made a hidden informal bond where she was happy to pay back with constructive feedback to those who did the same. F3’s behaviour was similar but for a different reason; her statement exposed the dilemma that students faced in giving honest feedback and the approach she used.

Some suggested making honest feedback mandatory may help.

“Made everyone go round in a circle, one thing they thought was good and one thing they thought could be better”. F3.

“Get a talking stick and hand it round and they can talk”. M6.

We can gather from these statements that students prefer to give feedback upon request from a teacher rather than spontaneously. It reflects the unwillingness of students to take such responsibility and a mandate like the above may take away that concern off students.

### Teacher contributions

Many students valued the teacher’s guidance and contribution to the discussion particularly where they couldn’t come to an agreement or where the group lacked understanding or knowledge.

“It was nice having someone to facilitate it…..if you had left the five of us in a room to watch the videos and talk about it, we would only say nice things about each other and probably come up with a load of questions that we didn't know the answers to”. F7.

“So with peer feedback will say you done it wrong but don't suggest a different way, teachers will know what the best way to do it or the smooth way to do it”. M1.

They also valued facilitator role being encouraging and understanding.

“It is nice to have a supportive environment where it is nice and not too nerve wrecking and not like an interrogation”. F7.

“…directs the feedback and stimulates the conversation because I think it is very easy for us to collapse into sort of sullen silence”. M4.

### Open space

The session provided a space for open discussion. The students took this opportunity to discuss the challenges they faced through day to day feedback in their clinical settings.

“Consultants don't really understand what exactly… or how it is you are meant to be doing things, especially with the examinations skills or clinical skills, how they learn doing, it is completely different to how you are expected to do in the OSCEs”. F7.

“Teachers….tend to have their own ways of doing things….. they are on their own specialties, so I found you just receive so many ways of doing things from different doctors”. M6.

Receiving dissimilar feedback has led to confusion and dissatisfaction among the students. Though the core of an examination or a skill is the same, clinicians may use different steps in their practice. The feedback session provided an opportunity for discussing the basis for variation in approach which enhanced clarity and widened their understanding.

“We are all just trying to work out the best way of doing the same thing and there are lots of different ways to do it”.

In addition to the learning gains, many students identified ‘social comfort’ as a group to some of their educational challenges, even the competitive student.

“Having a forum where you are encouraged to come forward means that we all feel, we can get some of our feelings off our chest. ……it can feel very isolating to spend so much time with every one gunning for themselves and doing their own thing and technique… we don’t have a lot of group teaching at all”. M4.

“It is better for bonding as a group, if everyone has made a little mistake and you can all kind of laugh at yourselves, and tell each other, ‘it is ok’”. F7.

## Discussion

### The role of video

Videos played a key part in the feedback session. An interesting finding was that some students were unaware of their mistakes on both communication and technical skills. Absence of insight into one’s short comings could be a reason for not acting on feedback and improving their performances. This agrees with previous studies [12, 23, 24] which reported improving both these skills using videos. The students’ statements also revealed an important factor in communication skills. It was not that they lacked compassion or empathy, but that they lacked the skill to express it. Therefore videos can be used to improve the feedback process. Videos also helped improve the confidence of those who were self-critical of their performances. As observed previously [14] many students voiced their initial anxiety towards videos but it was less among those who had previous exposures. It would be valuable to reprise the study with students who had undergone repeated exposure to video feedback.

### Competition and collaboration

This approach promotes collaborative learning, within a group of peers sharing knowledge, skills and understanding. Although some found this method challenging, they identified that mistakes were not unique to them which led to an increase in confidence. In our study, the students were motivated to work harder after seeing better performances rather than being distressed as described by Nilsen and Baerheim [13]. In Lindon-Morris and Laidlaw study [14], students feared of being judged negatively on their video performances by peers. This study showed that judgements on peers already existed prior to the feedback sessions. Many were supportive towards each other when they saw mistakes in other performances. This created a healthy environment for feedback where students were empathic and forthcoming with encouraging and helpful comments. However those students who are mainly driven by competition found these sessions challenging. They were reluctant to share their videos or knowledge within the group and did not recognise mutual benefits. Nevertheless competitors were proportionately very low, thus had little effect on the group learning.

### Open discussion

All, including competitive students valued the space provided by the teacher for open and free discussion. Many expressed perplexity at receiving varying feedback from different clinicians. The feedback discussions provided clarification whilst improving student understanding of the complexity of clinical practice. In addition to the educational role it also offered a social bonding within the group. Medical courses have plenty of teacher-led group teaching such as lectures or tutorials, but in this feedback session, students are guided in reviewing their videos, self-reflecting and providing feedback. This result in them being exposed to their strengths and weaknesses, but in a supported fashion. This may have helped students to break the silence and engage in conversation. Every student has instances where they can be proud of their performance and instances where they wish to avoid. Understanding of this common experience at a deeper level among group members may have fostered a special bonding.

### Feedback dilemma

The students’ expectations of constructive feedback on their performances did not match the perception on giving feedback. Some dwelled on positive responses without providing constructively critical comments, because of personal discomfort at discussing negative performances [25, 26] possibly contributed by our social and cultural attitudes, which favour a reluctance to express negative views on others. Our study uncovered another factor which was participants’ lack of insight into each other’s expectations. The students believed that peers might get upset when discussing areas for improvement. In contrary according to the data, participants expressed dissatisfaction when they did not receive constructive criticism. Changing the students’ attitudes from hurting or downgrading peers to helping and promoting learning through constructive feedback might be a key to successful feedback. This concept can be introduced by the teacher at the beginning of the session.

### Implication of the word: feedback

We could also gather from the students’ expectations that they have attended the session expecting to receive constructive feedback, so inclusion of the word feedback in the session title ensures that they come appropriately prepared. Nevertheless it may play a negative role in peer discussion when peers are requested to provide feedback to each other. We believe the word ‘feedback’ may have a socio-culturally created meaning which differs among the participants. For some, it meant negativity while some considered it as taking up a responsibility. McGarr and Clifford [15] stated that an implicit, historically and culturally formed relationship exists between teachers and students which each have established expectations of the other. Thus students may perceive giving feedback to peers as taking up a teacher’s role. Hence, replacing feedback with another word such as discussion where students have no connotation may be helpful to mitigate these issues, when introducing the students’ roles in the session.

### Teacher’s role: more than a facilitator

Success of the feedback session depends on the teacher’s contribution. Though students play a dominant role, the teacher provides the framework. It is not only the expert knowledge but guidance, encouragement and creation of a supportive environment for students to open up and engage in feedback discussion. Teacher’s feedback goes beyond the traditional one to one approach. For example, if a student’s misunderstanding surfaces when providing peer feedback, the teacher has the opportunity to address it.

The majority of published literature focuses on individual components of this approach; either video based self-reflection, peer feedback or teacher’s feedback, each of which has limitations, are reduced through combination. This study highlighted the benefits and challenges of this holistic approach on student learning through their perception.

We received overwhelmingly positive response from the student evaluation on this feedback approach. Therefore we have introduced this feedback session with each formative OSCE since this study. We have had only two instances with poor performance where we did not discuss the video in the group setting during the past three years.

### Potential applications

The feedback concept defined here could be introduced in different practical skills; communication skills such as history taking or breaking bad news, clinical examinations and practical skills. It can be adopted in different situations where feedback is necessary, in teaching, revision or formative assessments. Though any year group would be suitable to commence this feedback approach, initiating early in the course from their first year may lead to more learning gains.

### Limitations

Our sample size was small and may not have represented the whole cohort of students. The overarching challenge in this study was the students’ unfamiliarity with many components of the process. The recordings of their performance, watching their videos in a group, commenting on peers and responding in a group discussion were all novel or nearly novel for many participants. Therefore the benefits and challenges we identified may not be the same with a more experienced group. A further study exploring teacher’s perspective may be of value.

## Conclusions

This study demonstrated many potential benefits of this holistic feedback approach. Combination of video based self-reflection, learning from peers’ videos, peer discussion and teacher’s guidance and expert comments had a cumulative educational value. The opportunity to reflect on videos, both self and on others and the open discussion between peers and the teacher engage the student at a deeper cognitive level than the standard descriptive feedback. The teacher plays a crucial role, guiding the session and providing effective feedback according to students’ needs. The study also shed some light into the challenges of the session and helps developing a number of recommendations which may alleviate some issues (Box 1).

## Box 1 Recommendations

The teacher can play a significant role in making feedback a success by:

• Providing a clear introduction at the beginning with explanations on aims and learning outcomes may promote students’ participation. Students can understand their expected behaviour in the session and the value of it.

 • Elaborating on the feedback dilemma and giving an insight into students’ expectations of feedback.

 • Encouraging open discussion in other teaching and feedback sessions as it could improve student experience and confidence.

 • Avoiding use of the word ‘feedback’ or ‘peer feedback’ when introducing the role of the students in the open discussion.

 • Providing a short debrief at the end, discussing challenges of the current session with the view of making the next one better.

## Abbreviation

OSCE: Objective structured clinical examination
